# On the Relationship Between Pain Variability and Relief in Randomized Clinical Trials

**DOI:** 10.3389/fpain.2022.844309

**Published:** 2022-04-08

**Authors:** Siddharth R. Tiwari, Andrew D. Vigotsky, A. Vania Apkarian

**Affiliations:** ^1^Illinois Mathematics and Science Academy, Aurora, IL, United States; ^2^Center for Translational Pain Research, Feinberg School of Medicine, Northwestern University, Chicago, IL, United States; ^3^Departments of Biomedical Engineering and Statistics, Northwestern University, Evanston, IL, United States; ^4^Departments of Neuroscience, Anesthesia, and Physical Medicine and Rehabilitation, Feinberg School of Medicine, Northwestern University, Chicago, IL, United States

**Keywords:** pain relief, placebo response, pain variability, clinical trials, prognostic

## Abstract

Previous research reports suggest greater baseline variability is associated with greater pain relief in those who receive a placebo. However, studies that evidence this association do not control for confounding effects from regression to the mean and natural history. In this report, we analyzed data from two randomized clinical trials (Placebo I and Placebo II, total *N* = 139) while adjusting for the effects of natural history and regression to the mean *via* a no treatment group. Results agree between the two placebo groups in each study: both placebo groups showed negligible semi-partial correlations between baseline variability and adjusted response [*r*_sp_ (CI_95%_) = 0.22 (0.03, 0.42) and 0 (−0.07, 0.07) for Placebo I and II, respectively]. The no treatment group in Placebo I showed a negative correlation [−0.22 (−0.43, −0.02)], but the no treatment and drug groups in Placebo II's correlations were negligible [−0.02 (−0.08, 0.02) and 0.00 (−0.10, 0.12) for the no treatment and drug groups, respectively]. When modeled as a linear covariate, baseline pain variability accounted for <1% of the variance in post-intervention pain across both studies. Even after adjusting for baseline pain and natural history, the inability of baseline pain variability to account for substantial variance in pain response highlights that previous results concerning pain variability and treatment response may be inconsistent. Indeed, the relationship appears to be neither consistently specific nor sensitive to improvements in the placebo group. More work is needed to understand and establish the prognostic value of baseline pain variability—especially its placebo specificity and generalizability across patient populations.

## Introduction

Randomized clinical trials are the principal method by which researchers assess treatment efficacy. Although clinical trials can provide valid evidence of a treatment's average effect (relative to some control), they often fail to demonstrate meaningful drug effects relative to placebo. Some researchers have cited high “placebo response” as the main cause of these “failures” (1–4), suggesting it may be prudent to exclude “high placebo responders” prior to trial commencement.

Researchers have sought out correlates to predict pain relief in the placebo-treated group, which could then be used to exclude patients who would contribute to clinical trial “failure” *via* their “placebo response.” One of these identified correlates is pain variability, which has been shown to negatively correlate with subsequent pain relief in the placebo-treated group across several chronic pain conditions (5–7). In other words, patients with the greatest pain variability at baseline tend to have the greatest decreases in pain following placebo administration. This relationship is specific to pain relief in the placebo group in some (6) but not all studies (7), and even absent in others (8).

Although previous work demonstrates a relationship between baseline pain variability and pain relief, other factors such as regression to the mean and natural history can contribute to improvements in pain reports (6, 9). Indeed, previous studies have acknowledged, but have not accounted for, the influence of these factors on decreases in pain reports. In this work, we aim to improve our understanding of the prognostic value of baseline pain variability by adjusting for baseline pain, natural history, and regression to the mean. Since baseline variability is simple to collect and calculate [cf. neuroimaging and genetic traits that are also correlated with greater pain relief following placebo; e.g., (10, 11)], its prognostic value and (placebo-) specificity could be easily exploited in both trials and practice.

## Methods

### Datasets

This was a secondary analysis of two, previously published randomized, double-blind, placebo-controlled trials conducted by our research group at Northwestern University in Chicago, IL.

#### Placebo I

##### Overview

The purpose of this study was to investigate factors associated with analgesia in chronic pain patients who receive placebo (11).

##### Participants

Individuals had to be 18 years or older with a history of lower back pain for at least 6 months. This pain should have been neuropathic (radiculopathy confirmed by physical examination was required), with no evidence of additional comorbid chronic pain, neurological, or psychiatric conditions. Individuals had to agree to stop any concomitant pain medications and had to be able to use a smartphone or computer to monitor pain twice a day. Additionally, the enrolled patients had to report a pain level of at least 5/10 during the screening interview, and their averaged pain level from the smartphone app needed to be higher than 4/10 during the baseline rating period before they were randomized into a treatment group. Here, we include 20 participants from the no treatment group and 43 participants from the placebo group. We excluded the trial's drug group because its small sample size (*n* = 5). This group was also removed in previous analyses of this trial (11). The demographic characteristics of these patients are presented in Table 1.

**Table 1:** Demographic characteristics of Placebo I and Placebo II.

		**Age (SD), years**	**Women (%)**
Placebo I	No treatment (*n* = 20)	46 (13)	10 (50)
	Placebo (*n* = 43)	46 (12)	14 (33)
	All (*n* = 63)	46 (12)	24 (38)
Placebo II	No treatment (*n* = 11)	55 (10)	7 (64)
	Placebo (*n* = 32)	58 (10)	18 (56)
	Drug (*n* = 33)	53 (14)	12 (36)
	All (*n* = 76)	55 (11)	38 (52)

##### Pain Data

Data were collected using a custom pain rating phone app through which patients could rate their pain (0–10 numerical rating scale, NRS). Patients were asked to enter their pain 2 times/day over the course of the entire study. For the purposes of demonstration, here we averaged pain ratings within a single day.

#### Placebo II

##### Overview

The purpose of this study was to validate a prognostic model for classifying chronic pain patients based on their predicted improvement with placebo (12).

##### Participants

Individuals with chronic low back pain were recruited for this study. Patients must have had low back pain for at least 6 months, with or without symptoms of radiculopathy, a minimum VAS score of 5/10 at the screening visit, and a minimum average pain of 4/10 over a 2-week period prior to their first visit. Patients were randomized to no treatment, placebo, or naproxen. Here, we include 11 participants from the no treatment group, 32 participants from the placebo group, and 33 participants from the naproxen group. The demographic characteristics of these patients are presented in Table 1. One participant from the placebo group was excluded due to insufficient baseline data.

##### Pain Data

Data were collected using a custom pain rating phone app through which patients could rate their pain (0–10 NRS), as in Placebo I. Patients were asked to enter their pain 2 times/day over the course of the entire study. For the purposes of demonstration, here we averaged pain ratings within a single day.

#### Statistical Analysis

All analyses were performed using R (13). We built a single linear regression model for each study (2 in total), using pre-intervention pain (mean of the first 7 days in the pre-intervention period), group, and baseline pain variability (SD_baseline_, calculated as the standard deviation of the pre-intervention phase) as independent variables and post-intervention pain (mean of the last 7 days in the intervention period) as the dependent variable. In addition to these three independent variables, we included the interaction between group and SD_baseline_ (herein referred to as group × SD_baseline_ interaction) to isolate the effect of SD_baseline_ on post-intervention pain by group. The effects of group and the group × SD_baseline_ interaction were computed using modified backward contrasts, in which each group was compared to the previous group (placebo I: placebo vs. no treatment; placebo II: placebo vs. no treatment, drug vs. placebo) and no treatment was the intercept or reference group. This was done to compare the additive effect of placebo relative to no treatment and drug relative to placebo, meaning that the previous level controls the level succeeding it, thereby adjusting for natural history (since the no treatment group represents the natural course of pain), regression to the mean (through the pre-intervention score covariate and no treatment group), and placebo effects. Specifically, the following contrast matrices were used to compare differences between the two groups:


(1) $$C_{Placebo\;I}=\left[\begin{array}{c} 1\;0\\ 1\;1 \end{array}\right],\;C_{Placebo\;II}=\left[\begin{array}{ccc} 1 & 0 & 0\\ 1 & 1 & 0\\ 1 & 1 & 1 \end{array}\right].$$

The rows of these matrices denote the groups in each study (factors for no treatment in row 1, placebo in row 2, and drug in row 3) and the columns represent the weight of each parameter on that group. This is mathematically equivalent to dummy coding such that patients in the no treatment group receive a 0 for placebo and 0 for drug; patients in the placebo group receive a 1 for placebo and 0 for drug; and patients in the drug group receive a 1 for placebo and 1 for drug. These contrasts enabled us to isolate the effects of SD_baseline_ on post-intervention pain by group.

After obtaining the isolated effects, we calculated semi-partial correlations ($r_{sp}=\text{sgn}\left(t\right)\sqrt{\frac{t^{2}\left(1-R^{2}\right)}{df}}$, where *t* is the *t*-statistic of the effect of interest, *R*^2^ is the model coefficient of determination, and *df* is the residual degrees of freedom) between SD_baseline_ and post by group. Compatibility intervals (CI) for *r*_sp_ were calculated using the bias-corrected and accelerated bootstrap with 1,000 replicates. Data are depicted using adjusted effects (14).

## Results

**Figure 1:**
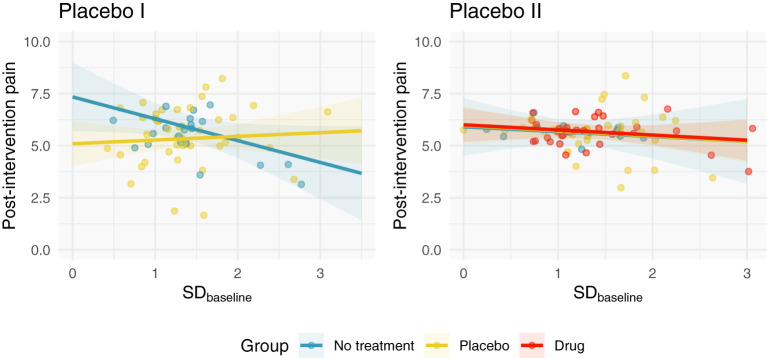
Adjusted post-intervention pain as a function of SD_baseline_ and group. We fit a linear regression to each study, which modeled post-intervention pain as a function of pre-intervention pain, SD_baseline_, and group. Here, we depict the relationship between SD_baseline_ and post-intervention pain after adjusting for pre-intervention pain. In Placebo I, the no treatment group has a weak negative correlation; the placebo group's SD_baseline_ is not correlated with post-intervention pain. In Placebo II, all groups demonstrate negligible correlations with SD_baseline_.

In total, 139 subjects were examined (63 subjects in Placebo I; 76 subjects in Placebo II). Figure 1 depicts the independent relationship between baseline pain variability and relief for each group after adjusting for pre-intervention pain, allowing each group to have a different effect of baseline pain variability. Model parameters and semi-partial correlations associated with adjusted group effects can be found in Table 2. Including SD_baseline_ in the models as a linear (not interactive) predictor increased Placebo I and Placebo II model *R*^2^'s by 0.01. Our observed effects were not strongly influenced by any individual patient (Supplementary Figure 1).

**Table 2:** Relationships between baseline pain variability and relief by group.

		**$\hat{\beta }$ (CI)**	***r_***sp***_* (CI)**
Placebo I	No treatment (*n* = 20)	−1.0 (−2.1, 0.0)	−0.22 (−0.43, −0.02)
	Placebo (*n* = 43)	1.2 (−0.1, 2.5)	0.22 (0.03, 0.42)
Placebo II	No treatment (*n* = 11)	−0.2 (−1.3, 0.8)	−0.02 (−0.08, 0.02)
	Placebo (*n* = 32)	0.0 (−1.2, 1.2)	0.00 (−0.07, 0.07)
	Drug (*n* = 33)	0.0 (−0.8, 0.8)	0.00 (−0.10, 0.12)

*Compatibility intervals (CI) are presented at the 95% level. $\hat{\beta }$ = unstandardized estimate from the marginal effect (expected change in post-intervention pain per unit increase in baseline pain SD), r_sp_ = semi-partial correlation coefficient*.

## Discussion

The purpose of this study was to quantify and isolate the relationship between baseline pain variability and post-intervention pain by group in two randomized, placebo-controlled clinical trials. Our work extends that of previous research by adjusting for the effects of regression to the mean and natural history *via* a no treatment control group (15–17). By assuming that pain relief is a linear combination of natural history, regression to the mean, the placebo effect, and the drug effect,^1^ we estimated the placebo- and drug-specific effects of SD_baseline_ on post-intervention pain.

Contrary to previous work, we observed negligible correlations in our primary model, with SD_baseline_ capturing ≲ 4% of the variance in post-intervention pain across groups in both studies. Of principal interest was the placebo-specific effect, which previous studies suggest is on the order of *r* ≈ −0.3 (6, 7). After adjusting for the no treatment group and pre-intervention pain, our placebo-specific estimates were incompatible with these previous estimates (Table 1). However, our results are consistent with the recent findings of Gillving et al. (8), who observed negligible correlations between variability and improvements in patients who received placebo. Together, these results suggest that SD_baseline_ may not be a strong, consistent, and “placebo-specific” predictor of pain relief across populations.

The magnitude and precision of our estimates were sensitive to the modeling strategy. When modeling the groups separately, our effect estimates were larger and had greater variance, especially in Placebo II (Supplementary Table 1; Supplementary Figure 2). Thus, modeling the groups separately produced CIs that encompass previously reported estimates, but our point estimates were still relatively small and did not favor the placebo group. These results are suggestive that modeling differences may partly explain the discrepancy between studies. Similarly, differences in populations and sample sizes are important factors to consider (5–8).

Studies validating a SD_baseline_-based prediction model are lacking. Nevertheless, the utility of SD_baseline_ for trial exclusion is dubious. Even if SD_baseline_ or some other variable was strongly predictive of pain relief following placebo, the removal of so-called “placebo responders” would also affect the active treatment group, especially since “placebo effects” are thought to be one component of active treatment effects. Finally, although removing “placebo responders” would theoretically improve treatment effect estimates, the observed treatment effect for such a study would answer a different question since the sample is conditioned on SD_baseline_. This may result in an optimistic, ecologically questionable estimate that may be unlikely to translate to the clinic.

Rather than trying to optimize treatment effect estimates in trials using peculiar exclusion criteria, researchers should optimize treatments and thus their effect estimates for the ecological patient population. After all, the goal of research is not to find large effects—it is to find large effects that will successfully translate and improve lives. Notwithstanding the limitations of conditioning on SD_baseline_ for trial inclusion, there are two salient issues to note concerning SD_baseline_ as a prognostic variable. First, since we and others (7, 8) did not observe consistent or placebo-specific effects, the strength and consistency of previously reported placebo-specific results deserves greater scrutiny and, hopefully, reconciliation (5, 6). Second, although multiple studies have observed relationships between baseline pain variability and pain relief, its prognostic value has only been estimated, not validated (19). Neither of these two points preclude SD_baseline_ from having value, however. SD_baseline_'s ability to capture variance in trial endpoints (5–7) indicates it may be useful to include as a covariate in trial analyses, which may serve to improve statistical efficiency and estimates of treatment effects (20).

## Data Availability Statement

The original contributions presented in the study are included in the article/Supplementary Materials, further inquiries can be directed to the corresponding author.

## Ethics Statement

The studies involving human participants were reviewed and approved by Northwestern University IRB. The patients/participants provided their written informed consent to participate in this study.

## Author Contributions

ST, AV, and AA conceived the research question. AA supplied data and resources. ST and AV performed analyses, drafted the manuscript, and created figures and tables. All authors read, edited, and approved the final version of the manuscript before submitting.

## Funding

This work was funded by the National Institutes of Health (P50DA044121-01A1). This material is based upon work supported by the National Science Foundation Graduate Research Fellowship Under Grant No. DGE-1324585.

## Conflict of Interest

The authors declare that the research was conducted in the absence of any commercial or financial relationships that could be construed as a potential conflict of interest.

## Publisher's Note

All claims expressed in this article are solely those of the authors and do not necessarily represent those of their affiliated organizations, or those of the publisher, the editors and the reviewers. Any product that may be evaluated in this article, or claim that may be made by its manufacturer, is not guaranteed or endorsed by the publisher.

## Footnotes

^1^Although additive assumptions are common, they are likely not true in the mechanistic sense (18). Yet, they may serve as reasonable first-degree approximations with high utility, allowing researchers to simplify their experiments (cf. full-factorial designs) and draw pragmatic conclusions.
