# Supplementary material for: On the Relationship Between Pain Variability and Relief in Randomized Clinical Trials
Source: Front Pain Res (Lausanne). 2022 Apr 8;3:844309. doi: 10.3389/fpain.2022.844309 (PMC9024103; doi:10.3389/fpain.2022.844309)
Supplement: Supplementary file 1 [file Data_Sheet_1.pdf]

Supplement

On the Relationship Between Pain Variability and Relief in Randomized Clinical Trials

Tiwari *et al.* (2022)

Frontiers in Pain Research

Supplementary Table 1. ***Modeling groups separately, with and without pre-intervention score as a covariate.***

			$\hat{\beta}$ **(CI)**	r_{sp} **(CI)**
**Within-group change score, no pre covariate**	**Placebo I**	No treatment (n = 20)	−1.0 (−2.1, 0.0)	−0.44 (−0.78, 0.05)
		Placebo (n=43)	0.3 (−0.6, 1.2)	0.11 (−0.16, 0.41)
	**Placebo II**	No treatment (n=11)	−0.2 (−0.8, 0.4)	−0.26 (−0.69, 0.25)
		Placebo (n=32)	−0.2 (−1.0, 0.5)	−0.11 (−0.46, 0.21)
		Drug (n=33)	−0.2 (−0.6, 0.2)	−0.21 (−0.66, 0.28)
**Within-group, with pre covariate**	**Placebo I**	No treatment (n = 20)	−1.1 (−1.9, −0.3)	−0.45 (−0.69, −0.09)
		Placebo (n=43)	0.2 (−0.6, 1.0)	0.07 (−0.25, 0.34)
	**Placebo II**	No treatment (n=11)	−0.2 (−0.9, 0.5)	−0.19 (−0.25, 0.06)
		Placebo (n=32)	−0.4 (−1.3, 0.5)	−0.16 (−0.28, 0.07)
		Drug (n=33)	−0.2 (−0.6, 0.3)	−0.12 (−0.18, 0.10)

Each group was modeled separately, with and without pre-intervention score as a covariate. These analyses are like previous analyses in the literature which may not control for pre-intervention score or model the groups separately. These relax the assumptions of, for example, equal regression toward the mean across groups. However, as a result, they fit more parameters.

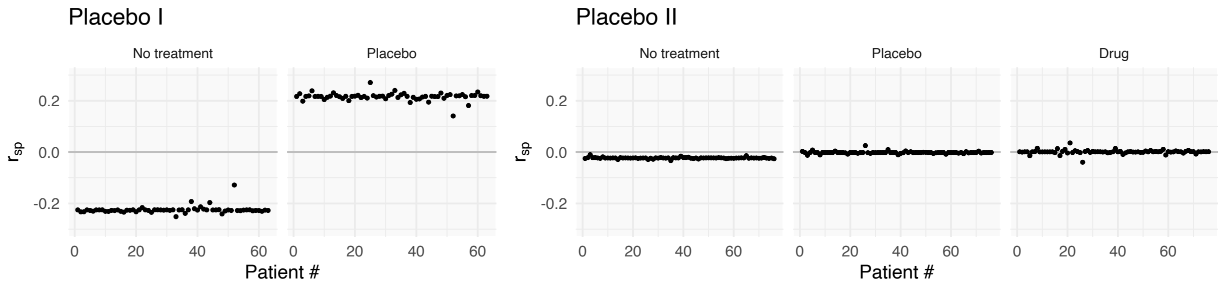

Supplementary Figure 1. ***Influence of individual patients on the semi-partial correlations.*** Each point represents the semi-partial correlation when patient *x* is removed from the analysis. This leave-one-out analysis reveals that in both Placebo I and Placebo II, leaving any single participant out does not strongly affect the results.

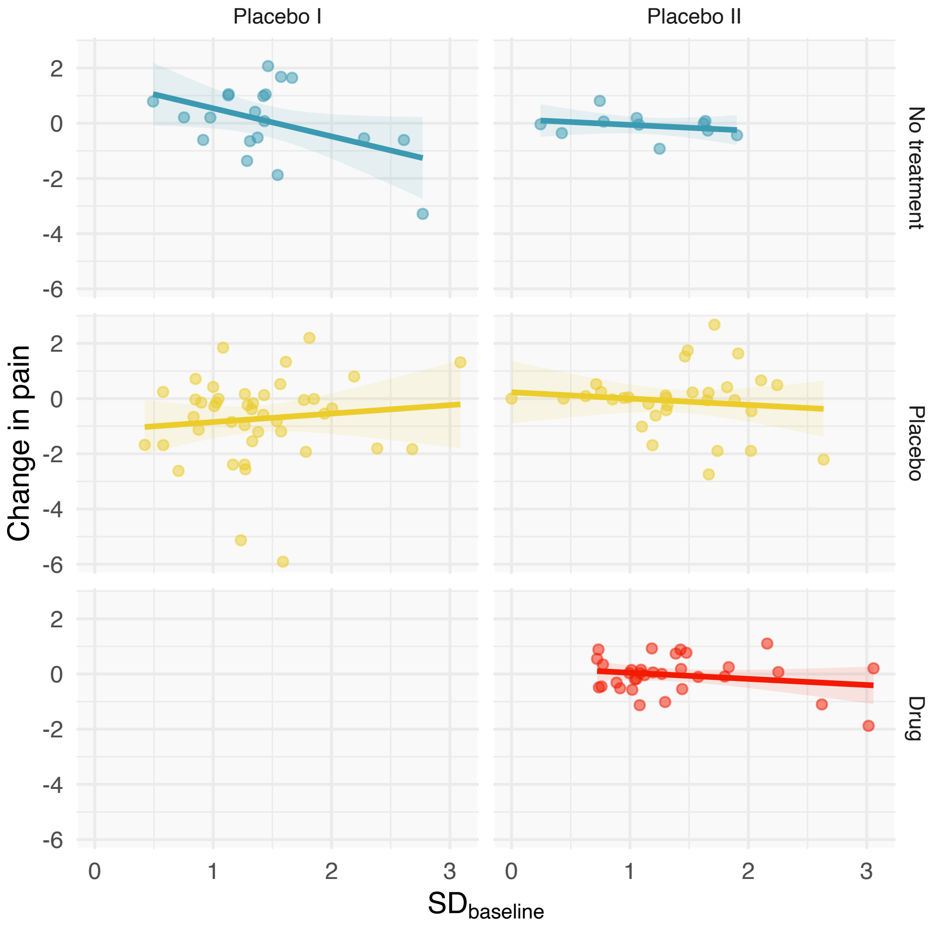

Supplementary Figure 2. ***Unadjusted relationships between baseline variance in pain scores and change in pain.***
